# Rhino-Orbital-Cerebral Mucormycosis: A Fatal Complication of Uncontrolled Diabetes Mellitus

**DOI:** 10.7759/cureus.1841

**Published:** 2017-11-13

**Authors:** Purva Bavikar, Varshil Mehta

**Affiliations:** 1 Department of Internal Medicine, MGM Medical College, Navi Mumbai, India

**Keywords:** mucormycosis, diabetes, complication, fungal infection, ophthalmic infection

## Abstract

Mucormycosis is a progressively invasive disease, with a fatal outcome, on late presentation. A 38-year-old female presented with diabetic ketoacidosis with right eye ptosis and a frozen globe without any signs of inflammation, externally. She underwent transnasal endoscopic debridement of paranasal sinuses and exenteration of the right eye. The histopathology specimen revealed the growth of mucormycosis. She was treated with intravenous (IV) amphotericin B, IV insulin, and extensive debridement surgery, but had an unfavorable outcome due to rapid mucor invasion to the brain.

## Introduction

Diabetes with rhino-orbital-cerebral (ROC) mucormycosis is a medical and surgical emergency. A high index of suspicion is critical for diagnosis, and early initiation of therapy often before confirmation of the diagnosis is necessary to optimize the outcomes [1]. Such patients require extensive debridement surgery, under cover of intravenous (IV) amphotericin B.

India has the second largest diabetic population globally (65.1 million) [2], with nearly 70% of these cases being those of uncontrolled diabetes [3]. On the basis of the data available from certain groups of patients, the prevalence of mucormycosis appears to be nearly 0.16% amongst diabetics and 1.2% amongst renal transplant recipients, with most of these cases manifesting as the ROC form [4,5].

It was observed that, India had an overall mucormycosis prevalence of 0.14 cases per 1000 population, with the prevalence rate ranging between 208,177 and 137,807 cases (Mean: 171,504; SD: 12,365.6; 95% CI: 195,777–147,688) and a mean of 65,500 (38.2%) attributable deaths per year [6].

## Case presentation

A 38-year-old female was admitted in the intensive care unit (ICU) for headache, fever, inability to open right eyelid and seizures. The patient had been well until approximately three weeks before admission, when headache developed, along with giddiness. The episodes occurred intermittently, initially localized to the frontal region, lasting for a few hours but later became diffuse and continuous. She also had occasional mild grade fever without chills since three weeks. She was unable to open right eyelid, which was associated with redness two days prior to admission. One hour before presentation, she had an episode of generalized tonic-clonic seizure. She was brought to the emergency department in a drowsy state. On arrival, she had another episode of generalized tonic-clonic seizure in the emergency room. There was no history of head trauma, limb weakness, vomiting, loss of consciousness, irrelevant talk, eye discharge, rash, bleeding manifestations.

She had undergone left third toe amputation two months back. The patient was a known case of diabetes mellitus for three years, and was taking oral hypoglycemic agents.

On admission, she was afebrile, vitally stable, with a pulse of 110/min, blood pressure of 130/70 mm Hg. She had non-healing ulcers over the right dorsomedial aspect of foot (1 x 1 x 0.5 cm) and 2 x 2 x 0.5 cm over the medial aspect of sole with pus discharging and her left third toe was amputated.

**Figure 1:**
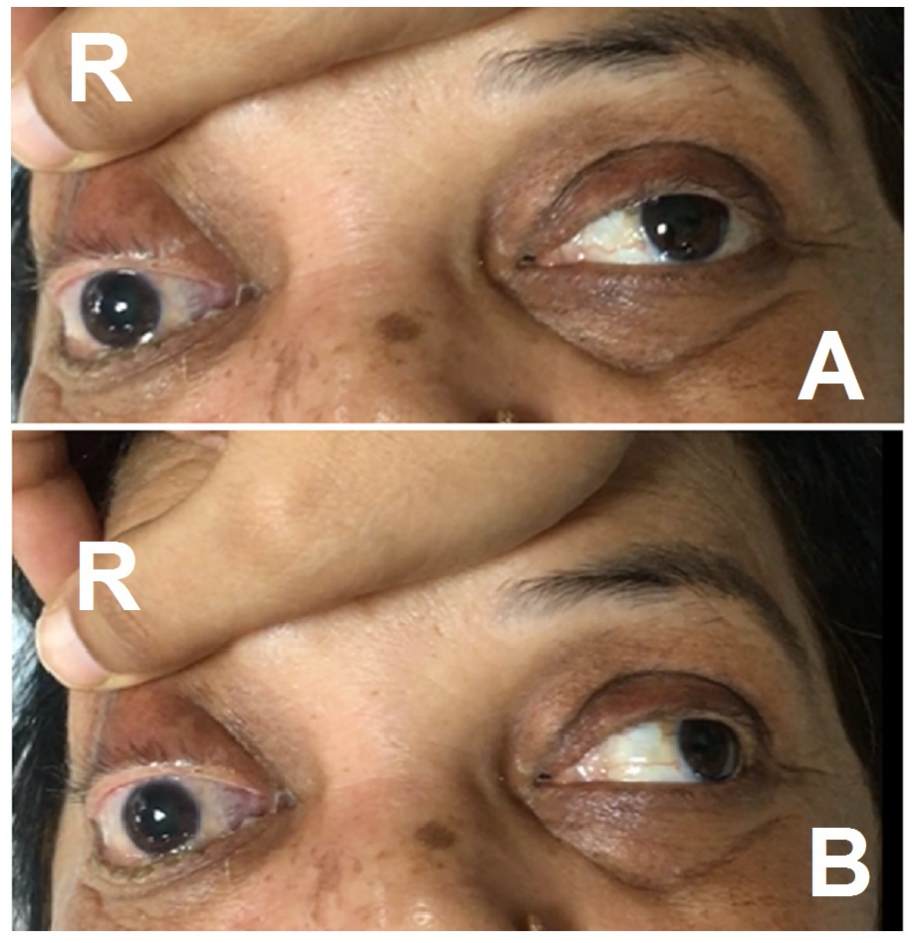
Right eye disorder on eye examination. (A) Fixed right eye. (B) Restriction on extra ocular movements.

On eye examination, she was unable to open her right eye and had mild maxillary tenderness. The right eye was fixed (Figure 1A) and had restricted (Figure 1B) extraocular movements. The pupil of the right eye was dilated, and the corneal sensation was absent with no perception of light. Her left eye was apparently normal. Systemic examination was normal.

On admission, her hemoglobin was 11.6 gm% with a total count of 22,670/mm^3^, predominantly neutrophils. Her blood sugar levels in the emergency room were ‘high’ (>500) on glucometer with ‘moderate’ urine ketones and ‘large’ urine sugars. The arterial blood gas (ABG) analysis was suggestive of metabolic acidosis. Serum sodium, potassium being 117 mEq/L and 4.8 mEq/L, respectively. The serum creatinine was 1.95 mg% with a blood urea nitrogen (BUN) of 24 mg/dl. Her hemoglobin A1c (HbA1c) was 15%.

A diagnostic nasal endoscopy revealed normal inferior turbinate, inflamed mucosa over middle turbinate and bulla ethmoidalis. There was lateralization of middle turbinate with distortion of normal anatomical features without any necrosis over soft tissue of turbinate. The blood staining discharge was removed and crust was sent for fungal smear and culture.

The magnetic resonance imaging (MRI) brain with venogram was suggestive of sinusitis involving right ethmoid, maxillary sinus with extension into the inferior and medial wall of right orbit into the superficial soft tissue overlying the right orbit and right nasal cavity. Increased bulk of right medial rectus and intra-orbital fat stranding was likely to be orbital cellulitis. The brain parenchyma was normal.

A differential diagnosis of diabetic ketoacidosis with either of the following – ophthalmoplegia secondary to diabetic-cranial nerve palsy or right orbit cellulitis or right rhino-orbital mucormycosis – was made. While her cultures were awaited, she was started empirically on IV cefoperazone sulbactam and IV metronidazole, with local eye drops – moxifloxacin, carboxymethyl cellulose. The diabetic ketoacidosis was treated with insulin infusion and IV fluids, with close monitoring of arterial blood gases and electrolytes. She was also started on tablet ecosprin and acetazolamide.

In the next three days, she developed gross facial asymmetry with generalized swelling over the right side of the face. There was periorbital edema appreciated more on the right side with proptosis and blepharoptosis. She had redness over right zygoma and infra-orbital region and crusting over lateral right ala of nose. Her left eyelid was swollen, with mild periorbital edema, but had the perception of light with intact extra-ocular movements in all directions of gaze and a positive corneal reflex.

A repeat diagnostic nasal endoscopy revealed extensive crusting, covering right inferior turbinate, middle turbinate region with a dull right lateral nasal, bulla ethmoiditis, maxillary region. The nasal crusts were removed and extensive debridement was done until the underneath soft tissue was seen.

**Figure 2:**
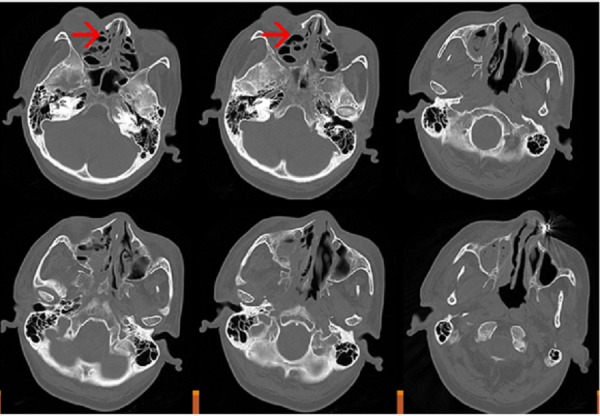
Contrast-enhanced computed tomography (CT) scan of paranasal sinuses is suggestive of soft tissue attenuation in right maxillary and frontal sinus.

Due to the evidence of such rapid progression, there was a high clinical suspicion of mucormycosis, hence conventional IV amphotericin B (1 mg/kg, i.e., 50 mg) was started on the third day and she was transfused with packed red blood cells (RBC). On day 4, we received cultures confirming *Staphylococcus aureus* (MRSA) growth on the nasal swab, with no fungal element seen on potassium hydroxide (KOH) mount. A contrast-enhanced computed tomography (CT) (Figure 2) of paranasal sinuses (PNS) was done which was suggestive of soft tissue attenuation in right maxillary and frontal sinus. The mucosal thickening was noted in bilateral ethmoid and left maxillary sinus and left frontal sinus. The air-fluid level was seen in the right sphenoid sinus, high-density content was noted in the base of the right maxillary sinus. The small osteoma was seen in the right ethmoid sinus. There was a hypertrophy of right inferior turbinate. The bony margins were intact. The osteomeatal unit of bilateral frontal and right maxillary sinus was blocked with soft tissue attenuation. The CT orbit suggested soft tissue attenuation in the right maxillary, right frontal, and right sphenoid sinus. The mucosal thickening was noted in bilateral ethmoid sinus and left maxillary sinus and left frontal sinus. No abnormality detected in orbits (Figure 2).

On day 5, she underwent endoscopic trans-nasal debridement of ethmoid and maxillary sinuses under general anesthesia. The black nasal eschar noted in the nasal cavity, ethmoidal sinuses and maxilla, was debrided after a nasal biopsy. The sample was sent for microbiological examination. Since there was no periorbital involvement seen during the procedure, the right eye was conserved. The patient tolerated the procedure well, was vitally stable hence, was extubated.

The microbiologist confirmed the growth of mucor on the nasal swabs, which were sent on day 1 of admission.

On the next day, post-operatively, the patient developed left-sided hemiplegia. A head CT was suggestive of normal brain parenchyma, and CT PNS was suggestive of chronic sinusitis in left maxillary, frontal and ethmoid sinuses and acute sinusitis in bilateral sphenoidal sinuses with an operative cavity in right frontal, maxillary and ethmoid sinuses.

**Figure 3:**
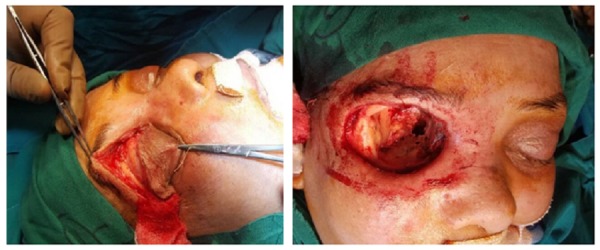
During exenteration of eye.

In view of further deterioration and grave visual prognosis, the decision of right eye exenteration under general anesthesia was made. After exenteration, when the globe was removed, fungal invasion to the bony part was noted which was extending, involving roof of orbit (frontal bone), cavernous sinus, nasal cavity and maxilla (Figure 3). Hence debridement of frontal sinus and medial orbital wall extending up to sphenoid sinus was done and necrosed tissue was removed and sent for microbiological and histopathological evaluation.

**Figure 4:**
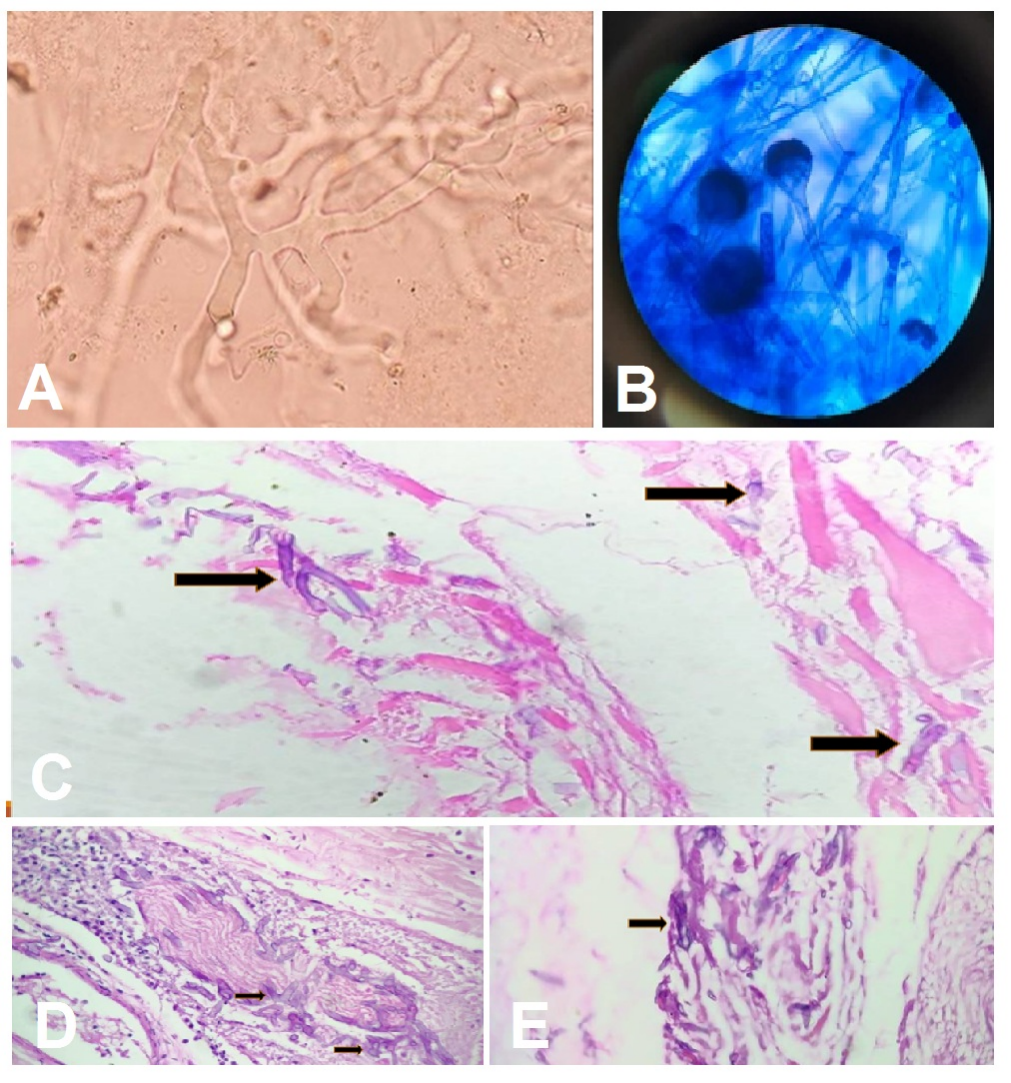
Histopathology slides. (A) KOH mount broad aseptate hyphae with branching. (B) Lactose phenol cotton blue mount thick walled, ribbon-like aseptate hyphae with wide angle branching and presence of sporangia containing sporangiospores. (C) Lateral rectus muscle. (D) Perineural tissue. (E) Periorbital tissue.

After the second surgery, the patient developed severe metabolic acidosis (pH 7.09, pCO2 33, HCO3 10, pO2 233, SO2 100), while on mechanical ventilation. Her routine labs (hemogram, renal function, liver function and coagulation) were all within normal limit. Despite all corrections, she developed cardiac arrest and expired nine hours after surgery. Two days later, we received all tissue culture reports (nasal biopsy) suggestive of mucormycosis (Figure 4A, 4B). The histopathology report of eye-ball contents showed the presence of fungus with extensive necrosis and lymphocytic infiltrate in the nasal mucosal tissue, lateral rectus muscle (Figure 4C), perineural areas (Figure 4D) and periorbital fat (Figure 4E). The fungi were broad, aseptate having ribbon-like hyphae with branching at 90⁰ angle (Figure 4A, 4B).

## Discussion

Mucormycosis is a highly invasive and relentlessly progressive, resulting in higher rates of morbidity and mortality as compared to many other infections. *Apophysomyces elegans* is the major cause of disease in India [1].

These fungi cause infection primarily in patients with diabetes or defects in phagocytic function (e.g., those associated with neutropenia or glucocorticoid treatment). The patients with elevated levels of free iron, which supports fungal growth in serum and tissues, are likewise at increased risk for mucormycosis. In iron-overloaded patients with end-stage renal failure, treatment with deferoxamine predisposes to the development of rapidly fatal disseminated mucormycosis; this agent, an iron chelator for the human host, serves as a fungal siderophore, directly delivering iron to the mucorales. Furthermore, the patients with diabetic ketoacidosis (DKA) are at high risk of developing rhinocerebral mucormycosis. The acidosis causes dissociation of iron from sequestering proteins in serum, resulting in enhanced fungal survival and virulence. Nevertheless, the majority of diabetic patients who present with mucormycosis are not acidotic, and, even absent acidosis, hyperglycemia directly contributes to the risk of mucormycosis by at least three likely mechanisms: (1) hyperglycation of iron-sequestering proteins, disrupting normal iron sequestration; (2) upregulation of a mammalian cell receptor (GRP78) that binds to mucorales, enabling tissue penetration (due to both a direct effect of hyperglycemia and increasing levels of free iron, which independently enhances GRP78 expression); and (3) induction of poorly characterized defects in phagocytic function [1].

The ROC mucormycosis continues to be the most common form of the disease [1]. The initial symptoms of ROC mucormycosis are nonspecific and include eye or facial pain and facial numbness followed by the onset of conjunctival suffusion and blurry vision. The fever may be absent in up to half of cases. White blood cell counts are typically elevated as long as the patient has functioning bone marrow. If untreated, infection usually spreads from the ethmoid sinus to the orbit, resulting in compromise of extraocular muscle function and proptosis, typically with chemosis. The onset of signs and symptoms in the contralateral eye, with resulting bilateral proptosis, chemosis, vision loss, and ophthalmoplegia, is ominous, suggesting the development of cavernous sinus thrombosis.

According to the multiple case series reported from tertiary care center in North India, the prevalence of different clinical types amongst mucormycosis cases is: ROC (48–55%), cutaneous (13–15%), pulmonary (7–17%), disseminated (5–12%), gastrointestinal (5–13%) and isolated renal (5–14%) [7].

A high index of suspicion is required for the diagnosis of mucormycosis. As mucorales are environmental isolates, definitive diagnosis requires a positive culture from sterile site.

Unfortunately, cultures are positive in fewer than half of cases of mucormycosis. The likely explanation for the low sensitivity of culture is that the mucorales form long filamentous structures that are killed by tissue homogenization - the standard method for preparing tissue cultures in the clinical microbiology laboratory. Thus, the laboratory should be advised when a diagnosis of mucormycosis is suspected, and the tissue should be cut into sections and placed in the center of culture dishes rather than homogenized [1].

The IV amphotericin B is the drug of choice for mucormycosis. The liposomal amphotericin B increases the efficacy of the drug facilitating prolonged administration of ROC mucormycosis without side effects [8]. Due to financial constraints, our patient received the conventional form of amphotericin B.

The treatment outcome is good when the disease is anatomically confined to the sinuses. The literature shows evidence of high success rates and survival benefit of treatment with local amphotericin B and sino-nasal debridement ± palatal resection, when the disease was restricted to sino-nasal or rhino-orbital mucormycosis. Once the patient has developed ROC mucormycosis, surgical debridement provides no survival benefit and mortality is inevitable.

A rapidly corrected underlying metabolic derangement is the most important criterion for considering conserving of orbits, even in the presence of total ophthalmoplegia and central retinal artery occlusion (CRAO) [9]. The exenteration is indicated in the advanced involvement of orbit.

The fungus spreads along the walls and the lumen of blood vessels, leading to necrotizing arteritis and ischemic necrosis of involved tissues [10], which results in poor penetration of systemically administered drugs. Hence, newer modalities need to be found, to treat patients with ROCM.

Lastly, management of ROC mucormycosis mandates a high index of suspicion in predisposed individuals. Given the urgency of administering therapy early, the patient should be treated while confirmation of the diagnosis is awaited.

## Conclusions

A case of DKA with mucormycosis requires prompt diagnosis, in order to prevent a fatal outcome. It is clinically challenging to treat rhino-orbital mucormycosis with intravenous amphotericin B alone. Nevertheless, the combination of treating DKA with insulin and IV fluids and mucormycosis with amphotericin B, adds to the complexity of the clinical case. The surgical debridement becomes mandatory as mucorales are rapidly invasive and progressive. It also needs to be emphasized that, treatment might depend mostly on high clinical suspicion, as the patient might be lost until cultures are awaited.
